# A rapid and standardized workflow for functional assessment of bacterial biosensors in fecal samples

**DOI:** 10.3389/fbioe.2022.859600

**Published:** 2022-08-22

**Authors:** Ana Zúñiga, Geisler Muñoz-Guamuro, Lucile Boivineau, Pauline Mayonove, Ismael Conejero, Georges-Philippe Pageaux, Romain Altwegg, Jerome Bonnet

**Affiliations:** ^1^ Centre de Biologie Structurale (CBS), INSERM U1054, CNRS UMR5048, University of Montpellier, Montpellier, France; ^2^ Hepatogastroenterology and Bacteriology Service at CHU Montpellier, University of Montpellier, Montpellier, France; ^3^ Department of Psychiatry, CHU Nimes, University of Montpellier, Montpellier, France

**Keywords:** synthetic biology, diagnostics, whole-cell biosensor, engineered bacteria, metabolite detection, gut microbiome

## Abstract

Gut metabolites are pivotal mediators of host-microbiome interactions and provide an important window on human physiology and disease. However, current methods to monitor gut metabolites rely on heavy and expensive technologies such as liquid chromatography-mass spectrometry (LC-MS). In that context, robust, fast, field-deployable, and cost-effective strategies for monitoring fecal metabolites would support large-scale functional studies and routine monitoring of metabolites biomarkers associated with pathological conditions. Living cells are an attractive option to engineer biosensors due to their ability to detect and process many environmental signals and their self-replicating nature. Here we optimized a workflow for feces processing that supports metabolite detection using bacterial biosensors. We show that simple centrifugation and filtration steps remove host microbes and support reproducible preparation of a physiological-derived media retaining important characteristics of human feces, such as matrix effects and endogenous metabolites. We measure the performance of bacterial biosensors for benzoate, lactate, anhydrotetracycline, and bile acids, and find that they are highly sensitive to fecal matrices. However, encapsulating the bacteria in hydrogel helps reduce this inhibitory effect. Sensitivity to matrix effects is biosensor-dependent but also varies between individuals, highlighting the need for case-by-case optimization for biosensors’ operation in feces. Finally, by detecting endogenous bile acids, we demonstrate that bacterial biosensors could be used for future metabolite monitoring in feces. This work lays the foundation for the optimization and use of bacterial biosensors for fecal metabolites monitoring. In the future, our method could also allow rapid pre-prototyping of engineered bacteria designed to operate in the gut, with applications to *in situ* diagnostics and therapeutics.

## Introduction

The human gut microbiota contains a large number of interacting species of bacteria, archaea, bacteriophages, eukaryotic viruses, and fungi which together create a complex ecosystem able to influence human physiology, pathologies, and behavior (Oliphant et al., 2019; Fan and Pedersen, 2020). The microbiome plays important roles in human homeostasis mostly related to metabolism (Smith et al., 2013). Multiple studies have linked abnormal-gut microbiota with altered metabolite profiles of patients with different diseases such as metabolic liver disease (Jiang et al., 2015; Schwenger et al., 2019), inflammatory bowel disease (Duboc et al., 2013; Nikolaus et al., 2017; Franzosa et al., 2019) as well as metabolic disorders like obesity and malnutrition (Ridaura et al., 2013; Smith et al., 2013; Sharon et al., 2014).

The analysis of feces metabolites has opened a new window on the complex interactions occurring within the gut (Wang et al., 2011; Patterson et al., 2016; Woting and Blaut, 2016; Jia et al., 2018). In the clinics, simple and quantitative tests enable measurements of fatty acid content or malabsorption of carbohydrates by analyzing the pH of feces (Caballero et al., 1983; Eherer and Fordtran, 1992). The detection of fecal proteins, particularly calprotectin, helps diagnose and monitor inflammatory bowel diseases (Manceau et al., 2017). In addition, bacterial infections of the gut can be detected using culture-based or molecular genotyping strategies (Karu et al., 2018). Finally, mass spectrometry coupled with liquid chromatography (LC-MS) has been successfully applied to measure the levels of metabolites in human feces allowing the prediction of key associations between diet and microbiome (Bjerrum et al., 2015; Bar et al., 2020). While LC-MS is increasingly used in clinical diagnosis and allows for general and precise metabolic profiling, it is still impractical and expensive for daily monitoring of metabolites (Seger and Salzmann, 2020). Additionally, heavy methods such as LC-MS are not deployable in the field or at home, restricting large-scale prospective routine monitoring of patients. The development of innovative point-of-care (POC) testing could enable real time clinical decision-making, eliminating requirements for specialists to perform and analyze the test. Therefore, new technologies are needed to support fast, field-deployable, and cost-effective detection of the metabolites produced by the microbiome in human samples such as feces.

Programmable bacteria present an attractive technology for engineering portable biosensor devices that could help address these challenges. A biosensor is composed of a biological sensing component, which recognizes a chemical or physical change, coupled to a transducing element that produces a measurable signal in response to the environmental change (Daunert et al., 2000). A wide number of bacterial biosensors have been engineered to detect different types of analytes by connecting natural transcriptional responses to different reporter genes (Chang et al., 2017; Hicks et al., 2020). Bacterial biosensors have significant potential in applications for medical diagnosis as they perform analyte detection with a robust response, high sensitivity, and can be optimized to detect molecules in complex media such as clinical human samples (Courbet et al., 2015; Watstein and Styczynski, 2017; Chang et al., 2021). Moreover, they are inexpensive and easy to manipulate and store. Synthetic biology has enabled bacterial biosensor improvement by providing a large number of standardized genetic parts, together with systematic strategies for organism engineering, resulting in biosensors with a higher specificity, able to detect molecules in a relevant range of concentration (Hicks et al., 2020).

In addition, synthetic biology has demonstrated the promising *in vivo* application of engineered bacteria for the treatment of diseases, including metabolic disorders (Nelson et al., 2021), infections (Daeffler et al., 2017; Riglar and Silver, 2018), and modulation of the tumor microenvironment (Guan et al., 2021). Characterizing and optimizing the sensing performance of these bacteria under physiological conditions could improve their *in vivo* applications and therapeutics abilities.

Here we developed a rapid workflow for functional assessment of bacterial biosensors in fecal samples. We characterized the sensing performance of five bacterial biosensors on fecal solutions of IBD patients. Three of the biosensors evaluated are based on cytosolic transcription factor systems: a biosensor responding to benzoate, based on BenR activator and the pBEN promoter (Libis et al., 2016; Zúñiga et al., 2020); a biosensor responding to anhydrotetracycline (aTc), based on TetR repressor and the pTET promoter (Lutz and Bujard, 1997; Courbet et al., 2015), and a biosensor responding to l-lactate, based on LldR regulator and the pALPAGA promoter (Zúñiga et al., 2021). The other two biosensors, TcpP/TcpH and VtrA/VrtC, correspond to transmembrane chimeric receptors for bile salts, activated via ligand-induced dimerization (Chang et al., 2021).

We have assessed the matrix effect of feces on their response, which changed depending on the type of biosensor and the target molecule. Using this method, we were able to detect exogenously added metabolites in presence of fecal solution. Even more, we detected endogenous bile salts in some samples, demonstrating that the performance of bacterial biosensors could be optimized for fecal metabolite detection and used as a monitoring strategy. In the future, this method could be used for rapid pre-prototyping of engineered bacteria designed to operate in the gut, with applications to *in vivo* diagnosis and therapeutics.

## Material and methods

### Strains

Details about all bacterial biosensor used in this study are provided in Supporting Information (Supplementary Table S1). All experiments were performed using the *E. coli* strains DH5αZ1 and NEB10β (New England Biolabs). The different biosensors were grown in LB media with corresponding antibiotics (kanamycin 25 μg/ml or chloramphenicol 25 μg/ml). The inducers were: anhydrotetracycline used at a final concentration of 200 nM, benzoic acid used at a final concentration of 100 μM, l-lactate used at a final concentration of 10 mM and taurocholic acid (TCA) and glycodeoxycholic acid (GDCA) used at different final concentrations. All chemicals used in this research were purchased from Sigma-Aldrich.

### Human feces samples collection

Feces samples from routine monitoring of IBD patients were obtained from the Hepatogastroenterology and Bacteriology service at Centre Hospitalier Universitaire (CHU) Montpellier (France), in accordance with ethics committee approval (# 202101009). About 100–120 mg of samples were collected by using a Copan Liquid Amies Elution Swab (ESwab^®^) Collection and Transport System (ESWABR1, Copan ITALIA S. p.A). This consisted in a screw-cap tube filled with 1 ml of ESwab™ buffer, a modified liquid Amies medium (Amies, 1967), and is widely used for clinical samples preservation during transportation, both in the hospital and in field experiments, making our protocol compatible with existing workflows (Perry, 1997; Gumede et al., 2017; Saliba et al., 2020). ESwab™ buffer contains sodium chloride, potassium chloride, magnesium chloride, calcium chloride, monopotassium phosphate, disodium phosphate, and sodium thioglycollate (used to maintain the reducing condition of the media, and avoid oxidations of metabolites), but it does not contain charcoal as the original. After resuspension in the buffer, samples were immediately stored at −80°C until use. All experiments involving feces were performed in a containment level 2 laboratory.

### Feces processing

Collected samples on ESwab™ buffer were defrosted and homogenized for 2 min by vortexing, then centrifuged at 4,000 rpm for 10 min in Eppendorf tubes. The supernatant was recovered in a new Eppendorf tube and stored at −20°C until use. Further processing by filtering was done by using a 13 mm diameter sterile syringe filter with a 0.45 µm or 0.2 µm pore size hydrophilic PVDF membrane (Millex-HV Syringe Filter, Millipore). The dilution of feces solution was done following the general mixing of volumes; 75 µl 2X LB medium, plus 1.5 µl of biosensor culture, plus 3 µl of inducer adjusted at the needed concentration and 70.5 µl of the feces solution diluted in ESwab™ buffer to have a final volume of 150 μl, as follow; for 10% final feces concentration: 15 µl of feces samples plus 55.5 µl of ESwab™ buffer, for 25% final feces concentration: 37.5 µl of feces samples plus 33 µl of ESwab™ buffer and for 50% dilution 75 µl of feces samples. See Supplementary Protocols for a step-by-step procedure.

### Functional characterization of bacterial biosensors in feces

Bacterial biosensors from glycerol stock were plated on LB agar plates supplemented with antibiotics and incubated at 37°C overnight. For functional characterization, three fresh colonies of each bacterial biosensor were picked and inoculated into 0.5 ml of LB with corresponding antibiotics and grown at 37°C for 16 h in 96 DeepWell polystyrene plates (Thermo Fisher Scientific, 278,606) sealed with AeraSeal film (Sigma-Aldrich, A9224-50EA) with shaking (200 rpm) and 80% of humidity in a Kuhner LT-X (Lab-Therm) incubator shaker. The next day, the cultures were diluted 1:100 into a final volume of 150 μl of LB supplemented or not with different dilutions of feces samples, and corresponding inducers, in 96-well plates, incubated at 37°C without shaking for 16 h, and analyzed by flow cytometry. Pooled or individual samples of fecal solution were used depending on the experiment. Pooled samples were used to avoid patient-specific matrix effect and describe the global effect of fecal solution in the biosensor performance. Individual patient samples, in contrast, were used to determine patient-specific variation and matrix effect, and to measure individual metabolites levels for bile salts. Pooled or individual fecal solutions were diluted with various volumes of ESwab homogenization buffer depending on the final target feces concentration. The cultures were incubated at 37°C for 16 h without shaking, with the goal of having the simplest protocol possible for future field application to metabolite detection. Next, cells were well mixed and 100-times diluted in 1X Attune Focusing Fluid (Thermo Fisher Scientific) before cytometry analysis (for more details on the method see Supplementary Figure S2). See Supplementary Protocols for a step-by-step procedure.

### Enzymatic assays for total bile salts and lactate quantification in feces

Total bile acids in feces were measured using Bile Acid Assay Kit (Sigma-Aldrich MAK309, Merck, France). The l-lactate concentration was measured using a l-lactate Assay Kit (Sigma-Aldrich MAK329, Merck, France). 20 µl of pre-treated and 0.4 µm filtered feces samples were used for each reaction. All measurements were performed in duplicate on two different days.

### Flow cytometry analysis

Flow cytometry was performed on an Attune NxT flow cytometer (Thermo Fisher) equipped with an autosampler and Attune NxTTM Version 2.7 Software. Experiments on Attune NxT were performed in 96-well plates with settings; FSC: 200 V, SSC: 380 V, green intensity BL1 488 nm laser, and a 510/10 nm filter. All events were collected with a cutoff of 20.000 events. A control cell-line of *E. coli* containing a reference construct was grown in parallel for each experiment. This *in vivo* reference construct has a constitutive promoter J23101 and RBS_B0032 controlling the expression of a superfolder GFP as a reporter gene in the plasmid pSB4K5. The cells were gated based on forward and side scatter graphs. The events on single-cell gates were selected and analyzed to remove debris from the analysis by using Flow-Jo (Treestar, Inc.) software. The gating strategy is depicted in Supplementary Figure S5.

### Encapsulation of biosensor in alginate hydrogel beads

BenR-pBEN biosensor was grown in LB medium with 25 μg/ml chloramphenicol at 37°C with shaking at 200 rpm for 16 h. Cells were then centrifuged at 4,000 rpm for 5 min and resuspended in fresh LB medium with 25 μg/ml chloramphenicol to an absorbance of 4 at 600 nm. A 5% w/v of alginate solution was prepared by dissolving medium viscosity alginate (Sigma-Aldrich A2033) in MilliQ water and autoclave sterilized. The liquid solution of the biosensor was mixed with the alginate solution at 1:1 ratio to reach 2.5% alginate and roughly 2 × 109 cells/ml. This mixture of alginate and bacteria was then dropped into a sterile 5% w/v CaCl2 solution (Sigma-Aldrich C1016) to form beads of 2 mm diameter. Beads were cross-linked for 5 min in CaCl2 solution. See Supplementary Protocols for a step-by-step procedure.

### Detection of benzoate by encapsulated BenR-pBEN biosensor in alginate beads

BenR-pBEN biosensor alginate beads and non-encapsulated biosensor with the same bacterial concentration were incubated with LB medium, chloramphenicol 25 μg/ml and pooled fecal solutions from four different patients at different percentages, containing or not exogenously added benzoate at 100 µM. Induction was performed in a black 384-well plate with a clear flat bottom (Corning, United States) at 37°C for 16 h without shaking. The ESwab™ buffer was used to adjust the different percentages of feces as described in the feces processing section. The fluorescence intensity of the reporter gene and the bacterial optical density were then measured using the Cytation3 plate reader (Biotek instruments). The experiment was done in triplicates in two different days. See Supplementary Protocols for a step-by-step procedure.

### Data analysis

The calculation of relative promoter units (RPUs (Kelly et al., 2009)) was done by normalizing the fluorescence intensity measurements of each biosensor according to the fluorescence intensity of the control cell-line *E. coli* harboring a reference construct. We quantified the geometric mean of fluorescence intensity (MFI) of the flow cytometry data and calculated RPUs according to the following equation:

### RPU= (MFIsample)/(MFIreference promoter) (1)

The goodness of fit and the EC50 for each data from bile acids biosensors set were calculated by applying non-linear regression using Agonist vs response-variable slope function using GraphPad Prism.

The fluorescence raw data from encapsulated BenR-pBEN biosensor was processed by subtracting autofluorescence and normalizing by the absorbance at 600 nm. Then the relative percentage of activity (RPA) of the biosensors was calculated using the following expression:

### RPA = 100-[(Fic-Fis)*100/(Fic)] (2)

Where Fis corresponds to the normalized fluorescence in presence of different % of fecal solution plus the inducer while Fic corresponds to the normalized fluorescence without fecal solution in presence of the inducer.

Statistical analysis was performed using GraphPad Prism unpaired t-student test and two-way ANOVA with Fisher’s LSD multiple comparisons test.

## Results

Fecal samples preparation for matrix effect assessment on bacterial biosensor performance.

**FIGURE 1:**
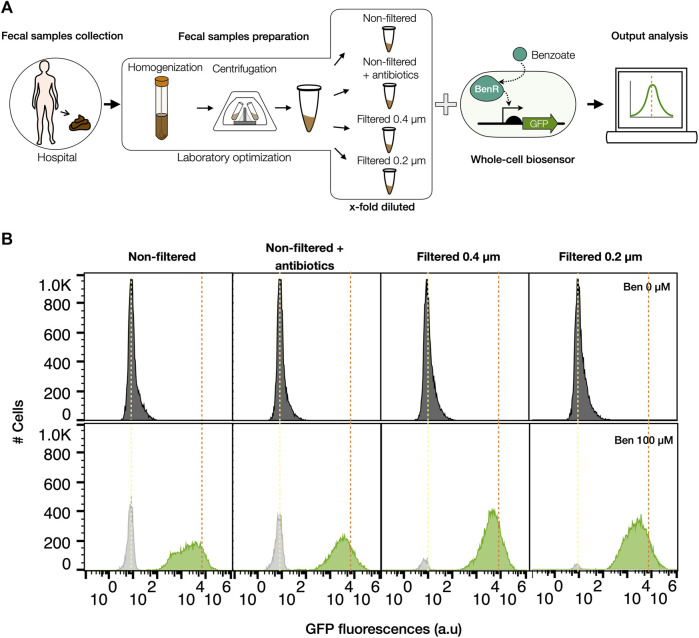
Human feces preparation to reduce matrix effects on bacterial biosensor performance. **(A)** Schematic workflow of the fecal matter preparation to assess matrix effect on benzoate bacterial biosensor performance. Samples were defrosted, homogenized, and centrifuged before evaluating the pBEN biosensor performance on different final fecal solutions. **(B)** Matrix effects of different fecal solutions on BenR-pBEN bacterial biosensor. Cells were induced with 100 µM benzoate (bottom) or non-induced (top) in presence of a 4-time diluted feces sample and incubated at 37°C without shaking for 16 h. Dark gray and gray histograms correspond to non-fluorescent populations. Green histograms correspond to the GFP-fluorescence population. Dotted lines represent the mean of the fluorescence produced by the biosensor growing in LB only, yellow: without benzoate; orange with 100 µM of benzoate. Each histogram is representative of two independent experiments measured by flow cytometry.

We first aimed to evaluate the matrix effect of feces on bacterial biosensors functionality. The first step in this process was to determine a simple and robust protocol to prepare a feces-derived solution (fecal solution thereafter) to which biosensors can be exposed. Our aim was to obtain from feces a sample that contains feces-derived metabolites yet is liquid so can be easily and reproducibly handled for testing with bacterial biosensors. The solution samples from 4 patients were recovered (see materials and methods for details) and pooled, to reduce the sample variability in the matrix effect estimation. Four treatment methods were applied to feces solution: 1) no filtration, meaning no change in the original microbial composition of the feces, 2) no filtration + antibiotics, to inhibit the growth of the endogenous bacterial microbiome during the experiment, 3) filtration at 0.45 µm and 4) filtration at 0.2 µm, to eliminate host-derived microorganisms in the samples (Figure 1A). Analysis of colony forming units (CFU) and absorbance at OD600 in LB medium confirmed growth of microorganisms only in fecal solutions without filtration (Supplementary Figure S1A), keeping a portion of the microbial composition able to grow under the conditions in which the bacterial biosensor performs. We evaluated the global matrix effect of pooled fecal samples by using a BenR-pBEN benzoate biosensor, based on a soluble regulator, due to the low concentration of this metabolite in human feces (Jenner et al., 2005; Muñoz-González et al., 2013; Gutiérrez-Díaz et al., 2018). We assessed the activity of the BenR-pBEN biosensor, with a limit of detection of 80 µM (Libis et al., 2016; Zúñiga et al., 2020), to sense exogenously added benzoate in presence of fecal solutions diluted at final concentration of 75%, 50%, and 25% vol/vol (Figure 1A) (see methods). We then evaluated the GFP fluorescence signal of the biosensor in the presence or in the absence of 100 µM benzoate (Figure 1B, Supplementary Figure S1). After 16 h of induction at 37°C, considerable inhibitory effects were observed in the benzoate biosensor output signal in 75% and 50% of feces (Supplementary Figure S1B). On the other hand, 25% of fecal solution allowed for a better sensing performance (Figure 1B). However, the fluorescence measurement was significantly lower in non-filtered samples compared to samples filtered with 0.45 and 0.2 µm (Figure 1B). In addition, a fraction of cells with no detectable fluorescence was observable in non-filtered feces induced with benzoate, probably corresponding to live or dead endogenous host microorganisms present in the matrix, reaching the 30% of the total population in the condition non-filtered but with antibiotics and more than 46% in condition with non-filtered fecal sample (Figure 1B and Supplementary Figure S1). Since the filtering of fecal samples at 0.2 µm did not show a significant improvement from the filtered at 0.4 µm, we chose the filtering at 0.4 µm to reduce the matrix effect of feces as a final protocol step. These results demonstrate that feces samples have significant inhibitory matrix effects on bacterial biosensors’ performance but by filtering and diluting fecal solutions, this effect can be reduced allowing bacterial biosensors to detect exogenously added metabolites.

**FIGURE 2:**
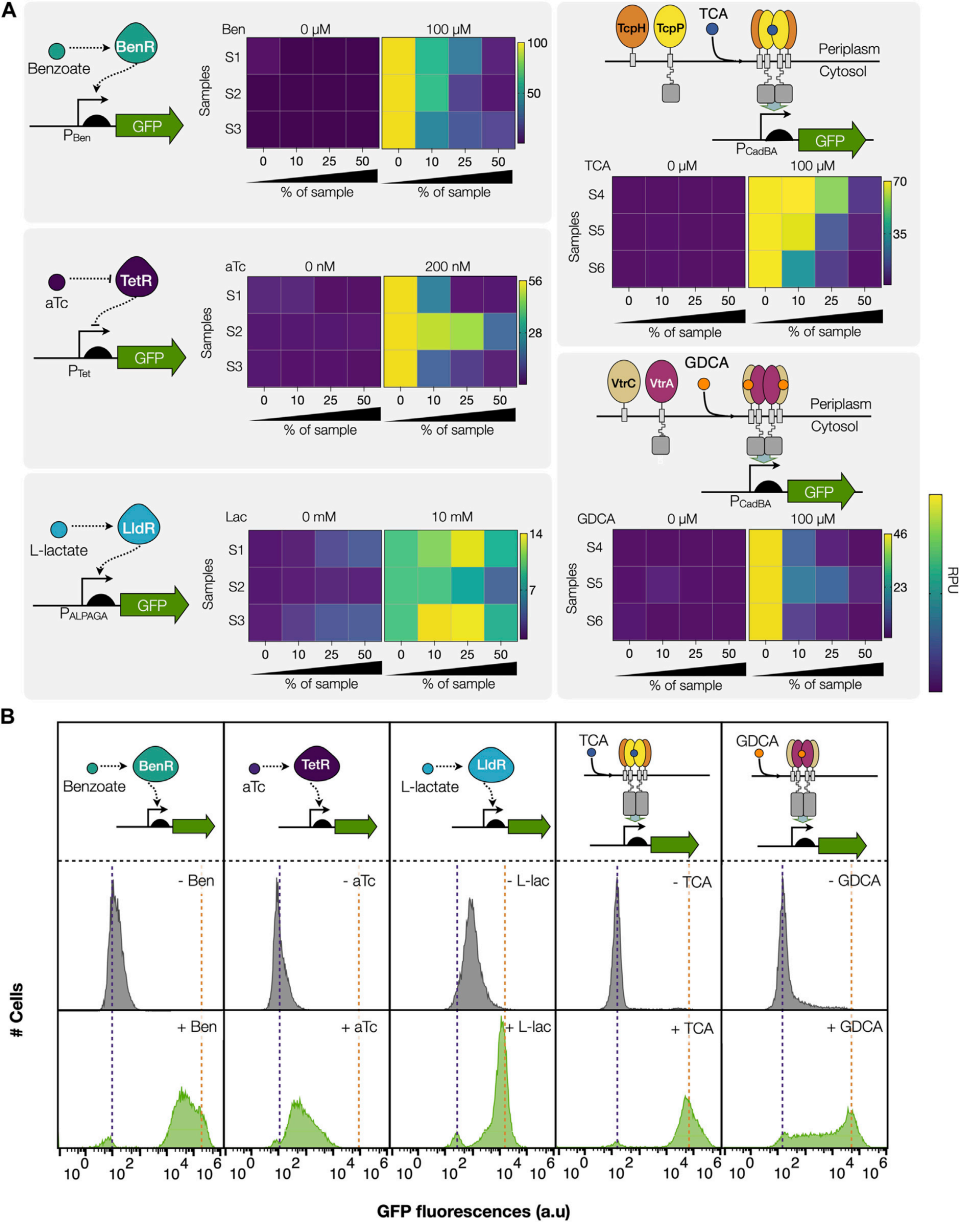
Performance of different bacterial biosensors in human feces. **(A)** Matrix effects of human feces samples on the performance of five different biosensors. Samples from different patients were used, S1-S6 represent the labels for the different patient samples. Biosensors at the top are based on cytosolic transcription factor systems: the BenR activator and the pBEN promoter; the TetR repressor and the pTET promoter and the LldR regulator and the pALPAGA promoter, induced with 100 µM benzoate, 200 nM aTc and 10 mM l-lactate, respectively. Sensors at the bottom are based on transmembrane receptors activated via ligand-induced dimerization, responding to bile acids: TcpP/TcpH to 100 µM TCA and VtrA/VtrC heterodimeric complex to 100 µM GDCA. Samples were used at final percentages; 10%, 25%, and 50% feces. Fluorescence intensities are expressed in relative promoter units (RPU) (see methods). The mean of three independent experiments performed in duplicate is plotted. The averages and standard deviations for these data are available in Table 1 and in Supplementary Data. For facilitating readability, note that the color scale was adjusted individually for each biosensor with the maximum value corresponding to values measured without the addition of fecal samples. **(B)** Representative histogram showing the fluorescence of the reporter gene for each biosensor expressed as a result of different induction conditions in 10% feces and measured by flow cytometry. Inducers; 100 µM benzoate, 200 nM aTc, 10 mM l-lactate, 100 µM TCA and 100 µM GDCA.

We then evaluated the sensing performance of different bacterial biosensors in presence of feces samples. We evaluated five different biosensors for: aTc; l-lactate; primary bile acids, taurocholic acid (TCA); secondary bile acids, glycodeoxycholic acid (GDCA); and benzoate. We evaluated the matrix effects on the sensing performance of these biosensors by adding feces samples at 2, 4, and 10-fold dilutions (50%, 20%, and 10% feces respectively, Figure 2A, see methods and Supplementary Figure S2). To observe the variability of the matrix effect we used solutions from six different patients (samples S1-S6). For TcpP/TcpH and VtrA/VtrC, in order to avoid interference from endogenous cognate ligands on the biosensor, the samples were chosen for their low content in total bile acids, as measured by an enzymatic assay, corresponding to samples S4-S6 (Figure 2A, Supplementary Table S2). All biosensors were able to detect their specific inducers in 10% feces samples. However, most of them failed to produce fluorescence in 50% of feces, except for lactate biosensors that showed a higher fluorescence when a higher concentration of feces was added. This was due to the endogenous presence of lactate in the samples (Supplementary Table S2), confirmed by enzymatic assay. Indeed, a feces sample with low measured lactate concentration (Figure 2A, patient S2) did not exhibit such increase in fluorescence. The different bacterial biosensors showed diverse degrees of sensitivity to fecal matrices (Figure 2B, Supplementary Figure S3, Table 1, and Supplementary Table S3). TetR was strongly inhibited (more than 50%), even in 10% feces. Interestingly, the VtrA/VtrC system exhibited a non-homogeneous response and was strongly inhibited too, although relying on a similar architecture to TcpP/TcpH, which was not significantly affected. This difference might be due to the fact that the behavior of TcpP was previously optimized by circuit tuning and directed evolution (Chang et al., 2021).

**TABLE 1:** Functional analysis of biosensors in human feces.

% of Sample	0%			10%			25%			50%		
Inducer	Leakage RPU	Max Fold Change	Max Swing RPU	Leakage RPU	Max Fold Change	Max Swing RPU	Leakage RPU	Max Fold Change	Max Swing RPU	Leakage RPU	Max Fold Change	Max Swing RPU
Benzoate	0.22 ± 0	104 ± 25	103 ± 14	0.21 ± 0	56 ± 13	56 ± 8	0.22 ± 0	23 ± 10	23 ± 5	0.2 ± 0	8.5 ± 6	8.2 ± 3
Anhydrotetracycline (aTc)	2.3 ± 1.3	27 ± 11	55 ± 10	2.1 ± 1.7	22 ± 10	42 ± 16	0.9 ± 0.6	14 ± 12	18 ± 10	0.8 ± 0.7	8 ± 5	6 ± 4
L-lactate	1.3 ± 0	8 ± 0.2	8.7 ± 0	2 ± 0.6	6 ± 1.2	10 ± 1	3 ± 0.4	4 ± 0.6	9 ± 1.8	3.4 ± 0.2	2 ± 0.4:	4 ± 1.4
Taurocholic acid (TCA)	0.6 ± 0.2	100 ± 23	68 ± 4	1.1 ± 0.6	48 ± 19	54 ± 12	0.7 ± 0.3	36 ± 21	26 ± 14	0.6 ± 0.2	8 ± 3	4.4 ± 2
Glycodeoxycholic acid (GDCA)	1.1 ± 0.5	43 ± 14	46 ± 8	1.4 ± 1.8	8.8 ± 6	11 ± 2	0.6 ± 0.4	10 ± 7	6 ± 3	0.5 ± 0.2	4.3 ± 2	1.6 ± 0.8

RPU, reference promoter units. The leakage RPU, measured in a non-induced state. The Max Fold change corresponds to the fold change between the induced state and the non-induced state. The Max Swing RPU corresponds to the subtraction of RPU between the induced stated and the non-induced state. The average of three independent experiments and standard deviations (±) are indicated. The concentration of inducers were: 100 μM Benzoate, 200 nM aTc, 10 mM L-lactate, 100 μM TCA, 100 μM GDCA.

Importantly, a clear patient-to-patient variability was observed for every biosensor, suggesting that feces matrix effects responsible for biosensor inhibition could be due to specific factors having varying abundance in different patients. These results show that several bacterial biosensors can operate in diluted fecal solution, yet have diverse sensitivity to fecal matrices, requiring case-by-case optimization.

### Detection of bile acids in human feces

Our next goal was to evaluate if by using our workflow for fecal processing bacterial biosensors could be used to detect endogenous metabolites in patients’ fecal samples. As a demo, we chose to assess bile salts, that are present in high concentrations in feces of patients with inflammatory bowel disease (IBD) (Jansson et al., 2009; Jacobs et al., 2016; Franzosa et al., 2019; Lavelle and Sokol, 2020). We assessed the detection of endogenous bile salts by using the TcpP/TcpH and the VtrA/VtrC biosensors.

**FIGURE 3:**
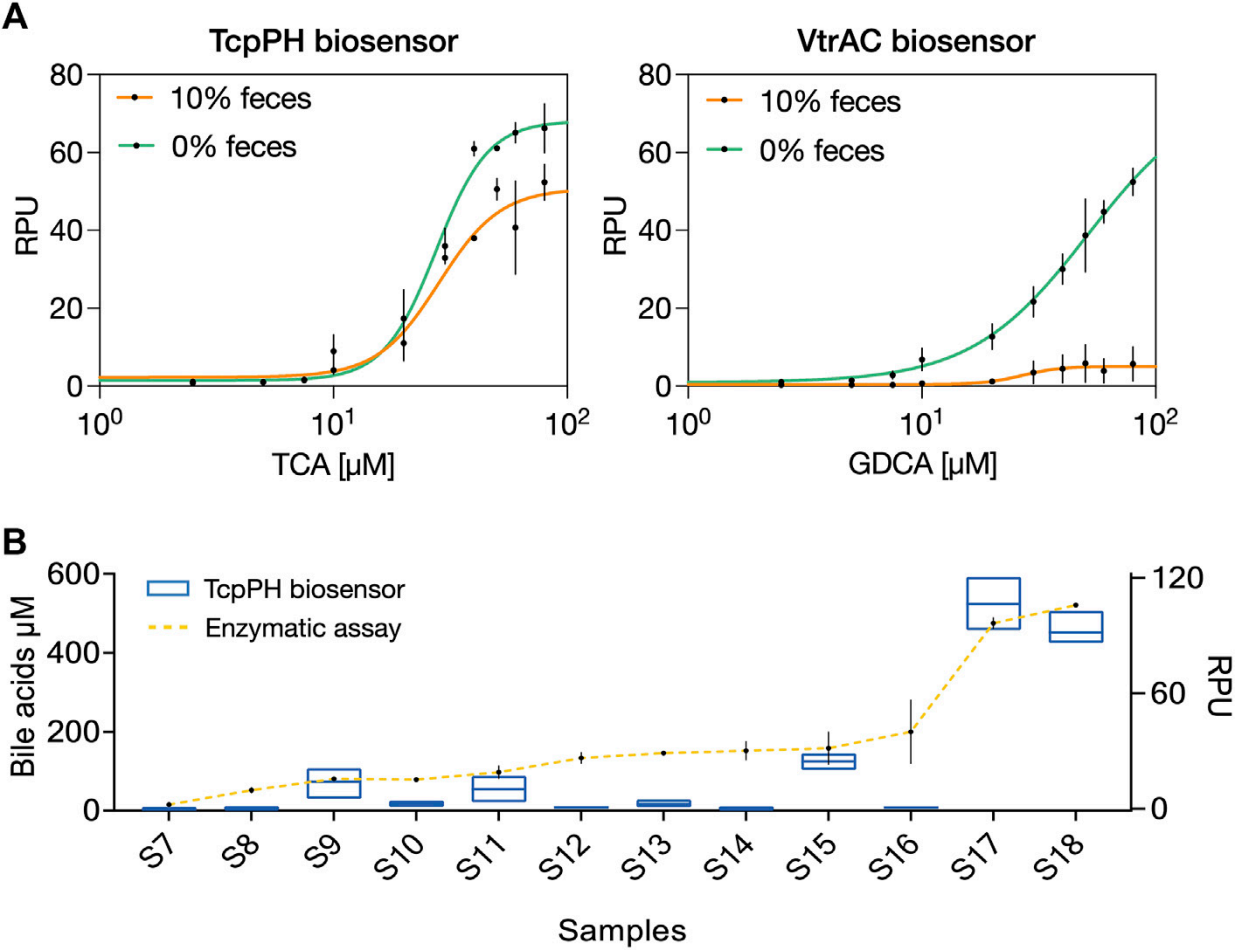
Bile acids detection in fecal solution from IBD patients. **(A)** The response function of TcpP/TcpH biosensor (left) and VtrA/VtrC biosensor (right) to spiked taurocholic acid (TCA) and glycodeoxycholic acid (GDCA) in the presence of 10-fold diluted feces (three different samples were pooled; samples S5, S6 and S7, see Supplementary Table S2). Data points correspond to the mean value of four replicates on four different days. Error bars: ±SD. **(B)** Comparison of total bile acid detection between TcpP/TcpH (blue square) biosensor, right axis, and enzymatic assay (yellow line) left axis. Samples were ordered according to their total bile salts concentration measured by the enzymatic assay. Data points correspond to the mean value of three replicates performed in duplicate on three different days. Error bars: ±SD.

We first evaluated the dose-response curve of TcpP/TcpH for TCA, and VtrA/VtrC for GDCA, in the presence or absence of 10% fecal solution (Figure 3A). We selected feces samples with low bile salts concentrations (S5-S7) to avoid interference from endogenous ligands. The TcpP/TcpH biosensor responded remarkably to spiked TCA in 10% feces solution, with high fold change and comparable limit of detection to the biosensor operating in the absence of feces. On the other hand, and as observed previously, the VtrAC biosensor was strongly inhibited by the presence of feces solution, confirming the need for additional optimization before performing detection of bile salt under these conditions. We then tested TcpP/TcpH for endogenous bile salts detection in feces solutions from 12 patients with IBD. We measured the total bile salts concentration with a commercial enzymatic assay (Sigma-Aldrich MAK309, Merck, France) (Supplementary Table S2) and used the same samples to evaluate the performance of TcpP/TcpH to measure endogenous bile salts (Figure 3B).

Samples exhibited different bile salts concentrations, all of them higher than 29 µM and two having very high bile salts concentrations (Samples S17 and S18, >500 μM, see Supplementary Table S2). The TcpP/TcpH system was able to detect bile salts in 5 different samples, with a strong response for samples S17 and S18. For the other samples, we observed a variable response from the biosensor (Figure 3B). These discrepancies might be due to several factors: 1) the biosensor LOD is too high for detecting low bile salts concentration. 2) Patient-specific matrix effects affect biosensor operation in some samples (e.g.S16) and 3) differential distribution of bile salts species depending on the patient: the enzymatic assay quantifies total bile salts, while the TcpP/TcpH system responds more specifically to primary bile salts, such as glycocholic acid (GCA), taurocholic acid (TCA), glycochenodeoxycholic acid (GCDCA), and taurochenodeoxycholic acid (TCDCA) (Chang et al., 2021). Importantly, in patients with IBD such as the ones in our study, the distribution between primary and secondary bile salts can greatly vary (Jacobs et al., 2016; Franzosa et al., 2019).

These results show that with an appropriate optimization of bacterial biosensors our workflow for fecal solution preparation could support the rapid detection of endogenous metabolites in human feces samples.

### Encapsulated biosensors in alginate show lower inhibitory matrix effect

**FIGURE 4:**
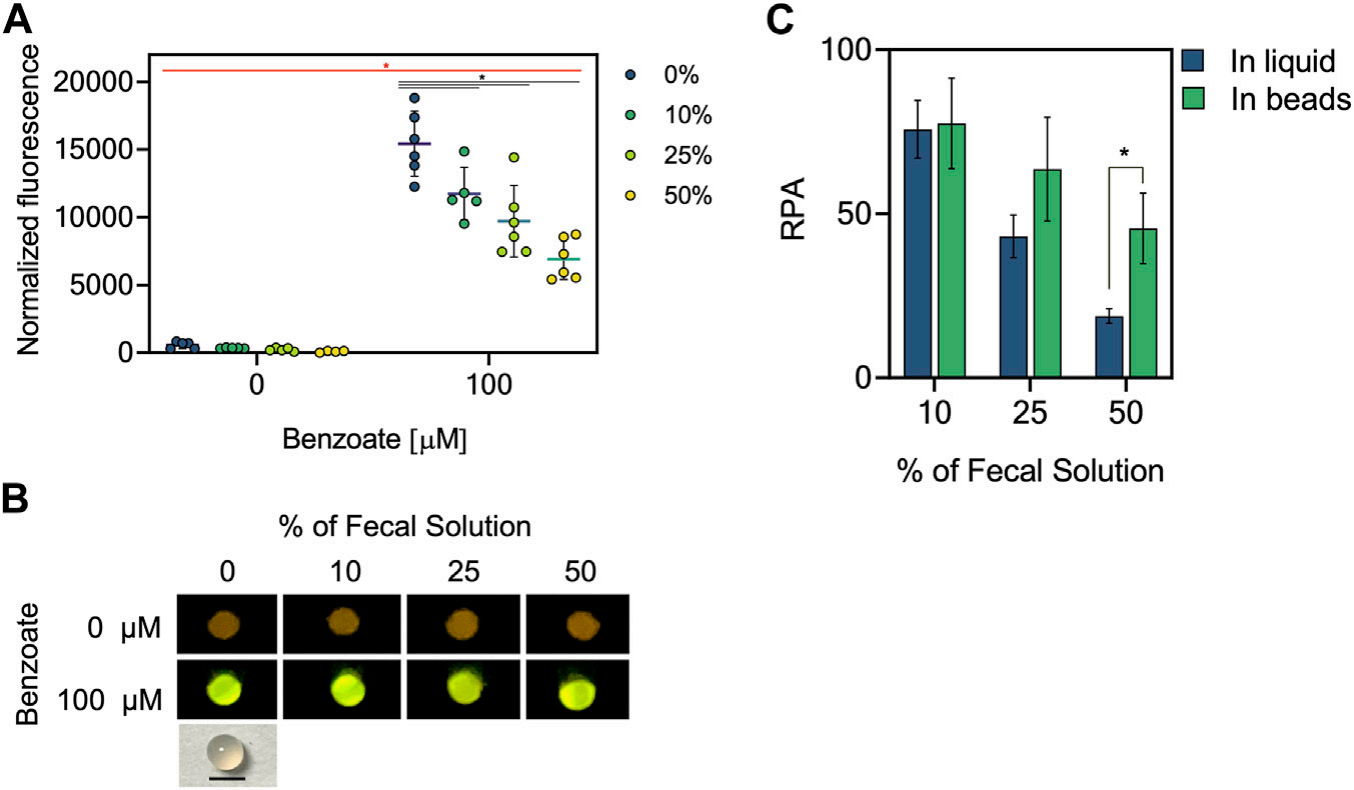
Matrix effect of fecal solution on the performance of encapsulated BenR-pBEN biosensor. **(A)** Normalized fluorescence of encapsulated BenR-pBEN biosensor on different percentages of fecal solutions. BenR-pBEN biosensor in alginate beads was induced or not with 100 µM benzoate in presence of pooled fecal solutions from four different patients at final percentages; 10%, 25%, and 50% and incubated at 37°C without shaking for 16 h. Fluorescence was measured by plate reader and normalized by absorbance at 600 nm. The mean value and standard deviation of beads analyzed in triplicate on two different days are plotted. *: *p*-value < 0.05 two-way ANOVA with Fisher’s LSD multiple comparisons test. The asterisk represents significant differences between non-induced versus induced condition (red) and non-fecal solution versus different percentages of fecal solution (balck). **(B)** Photograph of biosensor beads at the end of the sensing experiment performed (top) on different percentages of fecal solutions under blue light. The biosensor beads contained 2.5% alginate and measured 2 mm in diameter. Black bar: 2 mm. Error bars: ±SD. RPA was calculated as described in methods. An unpaired t-student test was performed. The asterisk represents significant differences between conditions. *: *p*-value < 0.05. **(C)** Relative percentage of activity (RPA) of encapsulated BenR-pBEN biosensor at final percentages of fecal solution; 10%, 25%, and 50%. Bars correspond to the mean of non-encapsulated (in liquid condition) and encapsulated (in beads condition).

Hydrogels beads have been studied as a desirable materials for encapsulating bacteria because they provide survival under stress conditions, allowing cell growth and biosensing functionality (Choi et al., 2013; Courbet et al., 2015; Li et al., 2017; Tang et al., 2021). In addition, hydrogel beads offer an attractive option for bacterial biocontainment (Li et al., 2017; Tang et al., 2021). To determine if encapsulation helps to reduce the inhibitory effect of fecal solutions we evaluated the relative percentage of activity (RPA) of BenR-pBEN biosensor encapsulated in alginate beads under different fecal solutions percentages (Figure 4). We optimized the protocol of bacteria encapsulation in alginate beads to get at least 1.3 × 10^9^ CFU/mL of live bacteria (Supplementary Figure S4) and ensure the biosensing process in fecal solution. Hydrogel beads containing the bacterial biosensor incubated in LB media for 16 h produced high fluorescence in presence of exogenously added benzoate under different concentrations of pooled fecal solution (Figure 4A). The high intensity of the fluorescence was observable under blue light (Figure 4B). We calculated the relative percentage of activity (RPA) and compared the sensing performance of biosensors in liquid culture versus encapsulated ones. We observed a significant performance improvement in beads in the presence of 50% of fecal solution compared to the liquid non-beads condition (Figure 4C), confirming a positive effect of beads encapsulation on reducing the inhibitory matrix effects of fecal solution.

## Discussion

In this work, we provide an optimized method to prepare fecal solutions for prototyping bacterial biosensors in human feces. We showed that a simple filtration step is enough to remove host microbes and reproducibly obtain a physiological-derived media retaining essential characteristics of human feces, such as matrix effects and endogenous metabolites (e.g. bile acids and l-lactate). We found significant inhibitory matrix effects of feces on the bacterial biosensors tested, although the robustness of the different biosensors varied. In addition, matrix effects varied significantly from patient to patient. This patient-to-patient variability could be due to host or microbiome-derived molecules that interfere with biosensor physiology. It is also possible that some medications inhibit the biosensors. Detailed knowledge of patients’ full clinical picture and current treatments will be essential to interpret the biosensor’s response. In all, the biosensors tested here are highly sensitive to fecal samples, and the optimal working conditions in our studies were general at a 10% feces sample dilution in liquid culture. However, we show that encapsulating bacteria in alginate leads improves the robustness of the biosensor against inhibitory matrix effects. We observed a significant improvement in the sensing performance of the benzoate biosensor in alginate beads compared to non-encapsulated cells in presence of 50% of fecal solution, without the need for any other optimization. This improvement may be due to two complementary factors; first, hydrogel beads can locally concentrate bacteria at a higher density, while providing water and nutrients to the cells and allows the diffusion of the inducer molecules to sense. Second, the alginate hydrogel itself offers a physical protection under stress conditions while conserving bacterial biosensing ability, as already shown in previous work by our group and others (Choi et al., 2013; Courbet et al., 2015; Li et al., 2017; Tang et al., 2021).

From our study, we can highlight several important points for future bacterial biosensor prototyping in feces: 1) because of biosensor-specific sensitivity to fecal matrices, case-by-case optimization of every new bacterial biosensor for operation in feces is required, 2) testing the biosensor over various individual samples coming from different patients is critical to obtain a bacterial biosensor working over a wide range of real-world conditions, and 3) encapsulation of biosensors can improve their performance in fecal solution by reducing their susceptibility to matrix effects. Using our workflow, these assays can be performed rapidly.

Interestingly, the TcpP/TcpH bile salts biosensor, that detects primary bile salts, was capable of detecting high concentrations of endogenous bile salts in samples from five different patients, in accordance with enzymatic measurements. Patients with IBD have altered fecal bile salts profiles (Duboc et al., 2013; Torres et al., 2018; Lavelle and Sokol, 2020), with lower levels of secondary bile salts but higher levels of primary (Jansson et al., 2009; Jacobs et al., 2016; Franzosa et al., 2019). For other samples, data was not in accordance with the enzymatic measurements, possibly because of different, patient-specific bile salts profiles, some bile salts not being detected by our bacterial biosensor. A more definitive answer could be provided in the future by using LC/MS to determine the exact bile salts species distribution in each sample. Nevertheless, as a proof of concept, these data demonstrate, for the first time to our knowledge, the possibility of using bacterial biosensors to detect endogenous metabolites in human feces.

How could bacterial biosensors operating in fecal samples be optimized in the future? First, other reporters having a higher signal-to-noise ratio, such as luciferase, might be evaluated. Yet, unless using the luxCDABE operon, which has lower performance, optimized luciferase systems such as nanoluc, while providing a lower limit-of-detection, work better after cell lysis, which would complicate the assay protocol (Lopreside et al., 2019). Second, amplifying genetic devices such as recombinase switches or hrp transcription factors might help combat matrix effects and enable operation at higher concentrations, thereby supporting lower limits of detection (Courbet et al., 2015; Wan et al., 2019). Recombinase-mediated inversion or excision could also allow *post facto* analysis of biomarker presence through DNA sequencing or PCR (Courbet et al., 2015; Wan et al., 2019). Furthermore, an appropriate coating on hydrogel beads complemented with genetic devices biocontainment (e.g. auxotrophs) (Steidler et al., 2003; Chan et al., 2016; Moya-Ramírez et al., 2022) could not only allow for a better performance of the biosensor on feces but also prevent bacteria leakage in the environment allowing the deployment of these biosensors as a point-of-care metabolite monitoring device.

**FIGURE 5:**
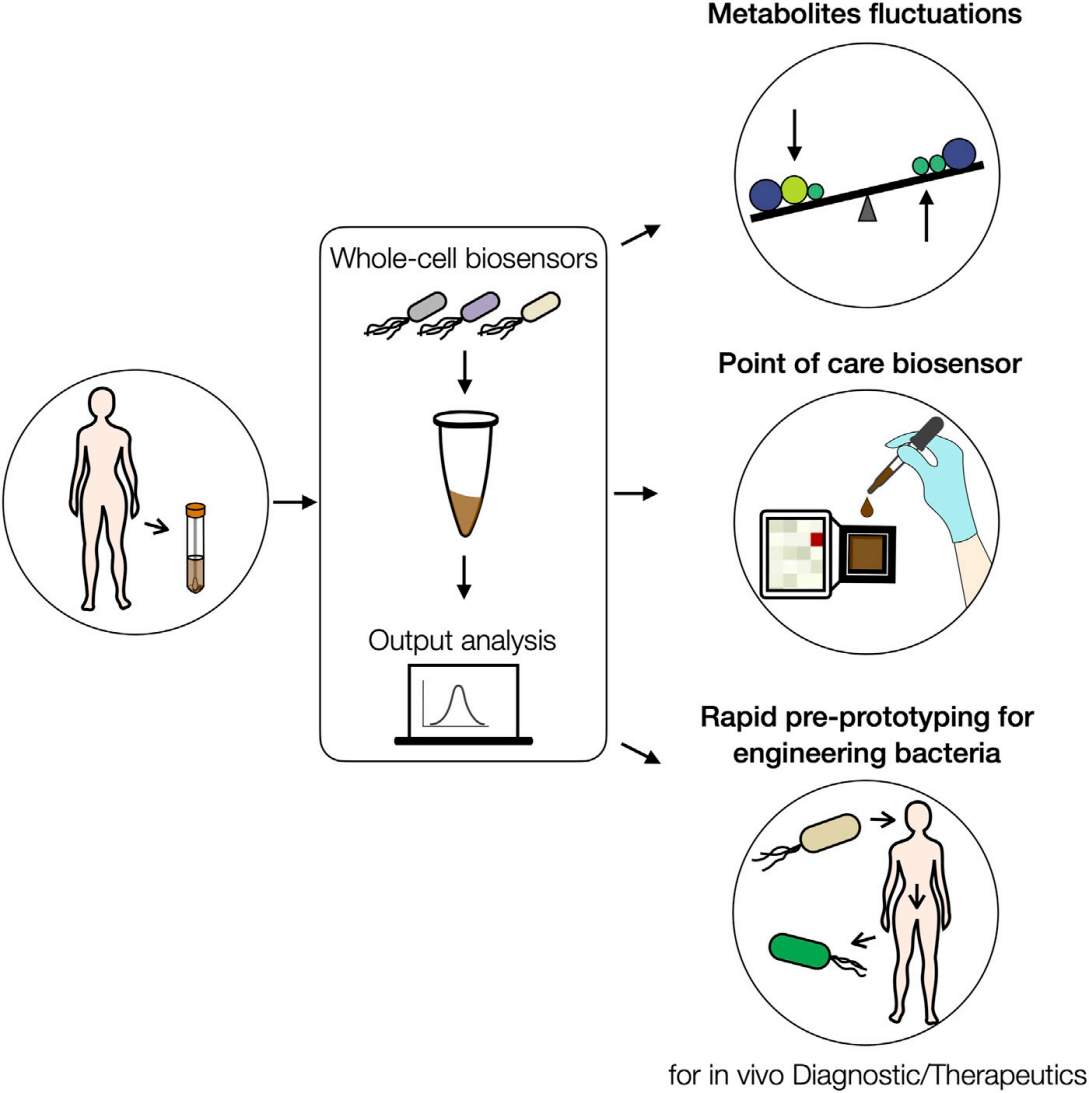
Summary of future applications for rapid prototyping of biosensors and metabolites detection in human fecal samples. By applying this method, human feces could be collected and homogenized at the hospital, and directly used to measure the levels of targeted metabolites by using bacterial biosensors. These measurements will help to understand metabolite fluctuations under a particular diet intake, for example. Similarly, this strategy would allow the engineering of point-of-care biosensors for performing medical diagnostics by measuring a set of targeted metabolites in feces. Finally, this prototyping approach could also support the engineering of therapeutic bacteria by allowing a fast characterization and optimization of their sensing performance closer to physiological conditions in fecal matrices.

The method shown here could be performed on a lab-on-chip device enabling successive feces samples filtration, dilution, and sensing assay in an automated manner (Wu et al., 2017, 2018; Arshavsky-Graham and Segal, 2020). Such devices would open the door to field-deployable, point-of-care gut metabolite detection either for diagnostics or epidemiological purposes (Figure 5).

Finally, another potential and compelling application of our method is its use for rapid and simple prototyping of engineered “smart” gut probiotics. Engineered bacteria have recently been developed to detect and/or treat many pathological conditions such as inflammation, diabetes, phenylketonuria, hyperammonemia, and cancer (Isabella et al., 2018; Riglar and Silver, 2018). As of now, these strains have been evaluated in animal or in cellular co-culture models (Mimee et al., 2016; Daeffler et al., 2017; Taketani et al., 2020; Nelson et al., 2021). While providing valuable information, these models present limitations in terms of time, physiological relevance, and amenability to screening. The use of human fecal samples could complement these approaches by providing a fast and efficient method to assess the matrix effects of fecal matter on bacterial sensors and therapeutics and optimize their behavior.

## Data Availability

The original contributions presented in the study are included in the article/Supplementary Material, further inquiries can be directed to the corresponding author/s.
