# Supplementary material for: A rapid and standardized workflow for functional assessment of bacterial biosensors in fecal samples
Source: Front Bioeng Biotechnol. 2022 Aug 22;10:859600. doi: 10.3389/fbioe.2022.859600 (PMC9444133; doi:10.3389/fbioe.2022.859600)
Supplement: Supplementary file 1 [file DataSheet3.pdf]

Supplementary Materials for

A rapid and standardized workflow for functional assessment of bacterial biosensors in fecal samples.

Ana Zuñiga[1], Geisler Muñoz[1], Lucile Boivineau[2], Pauline Mayonove[1], Ismael Conejero[3], Georges-Philippe Pageaux[2], Romain Altwegg[2], and Jerome Bonnet[1]*

Affiliations:

[1] Centre de Biologie Structurale (CBS). INSERM U1054, CNRS UMR5048, University of Montpellier, France.

[2] Hepatogastroenterology and Bacteriology service at CHU Montpellier, France.

[3] Department of Psychiatry, CHU Nimes, University of Montpellier, Montpellier, France.

* to whom correspondence should be addressed: jerome.bonnet@inserm.fr

Supplementary Figures S1-S5

Supplementary Tables S1-S3

Protocols S1-S2

Supplementary Figure S1

A

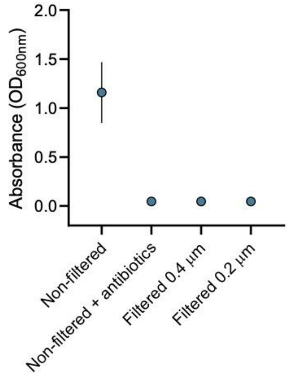

Fecal solution	CFU
Non-filtered	**1.6 x 10^5**
Non-filtered + antibiotics	–
Filtered 0.4 µm	–
Filtered 0.2 µm	–

B

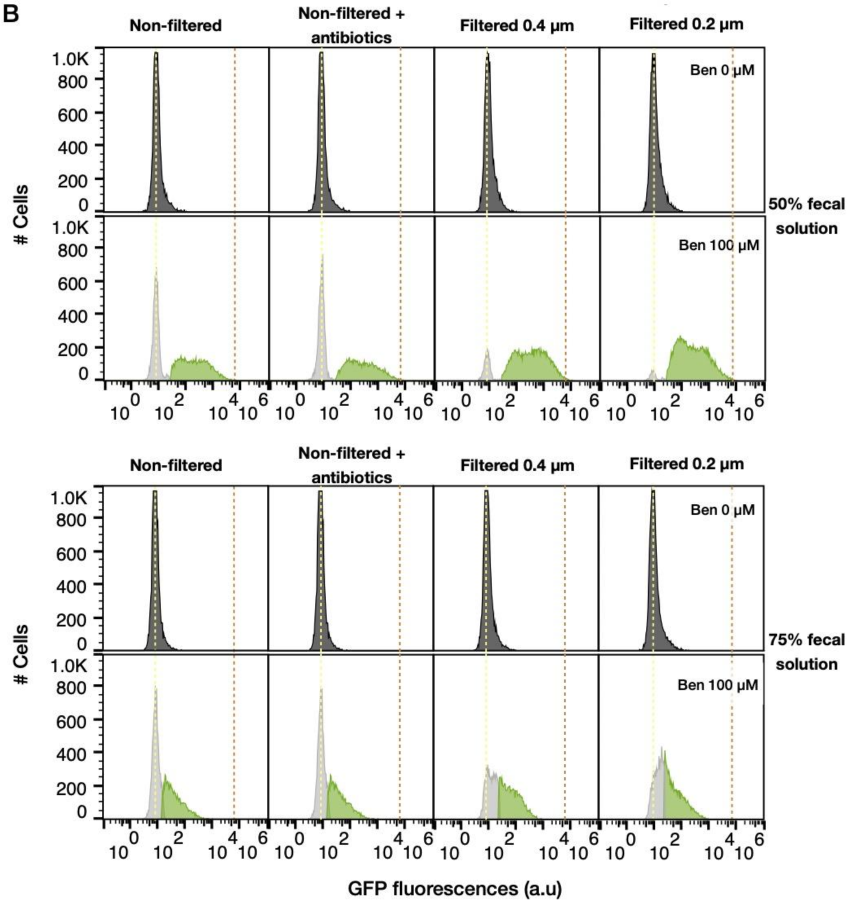

Supplementary Figure S1. **Optimization of human fecal solutions for a low matrix effect.** Samples were defrosted, homogenized and centrifuged before evaluating pBEN biosensor performance on different pre-treated feces. (A) Analysis of absorbance at OD600 (left) and colony forming units (CFU) (right) of fecal solutions. Feces were diluted 2-fold on LB medium and incubated at 37 °C without shaking for 16h, before absorbance (OD_{600}) measurement and colony count. (B) Cells corresponding to pBEN biosensor were induced or not with benzoate 100 µM and incubated at

37 °C without shaking for 16h. Feces were diluted 2- and 1.3-fold on LB (50% and 75%, respectively). Dot lines represent the mean fluorescence produced by the biosensor growing in LB only, purple without benzoate, orange with 100 μM of benzoate. Each histogram shows fluorescent reporter genes expressed as a result of different induction conditions. Each histogram is representative of two different experiments measured by flow cytometry.

Supplementary figure S2

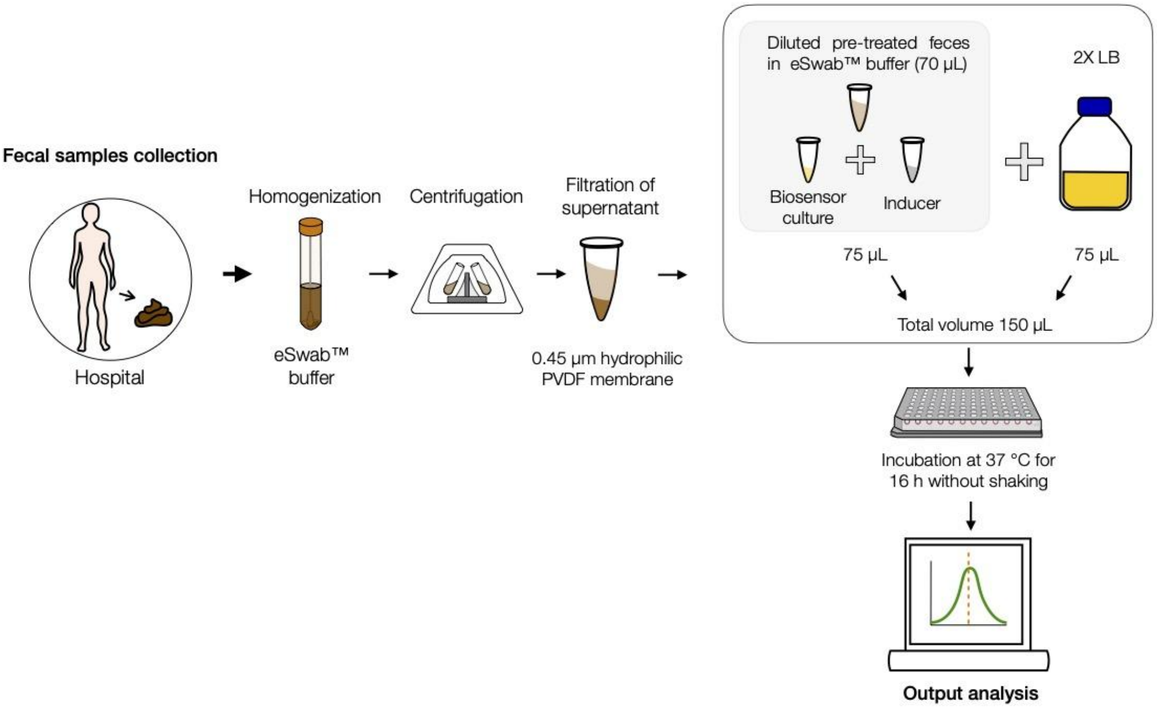

Supplementary figure S2. Methodology for metabolites detection by bacterial biosensors in fecal samples. Samples collected at the hospital are homogenized in the commercial ESwab buffer, then centrifuged for 10 min at 8.000 rpm, the supernatant collected and filtered by 0.45 µm hydrophilic PVDF membrane filter. Next, these pre-treated samples are mixed with 75 µL of 2X LB medium, ESwab homogenization buffer, biosensor culture and inducer until a final volume of 150 µL. The cultures are incubated at 37 °C without shaking for 16h, before cytometry analysis.

Supplementary figure S3

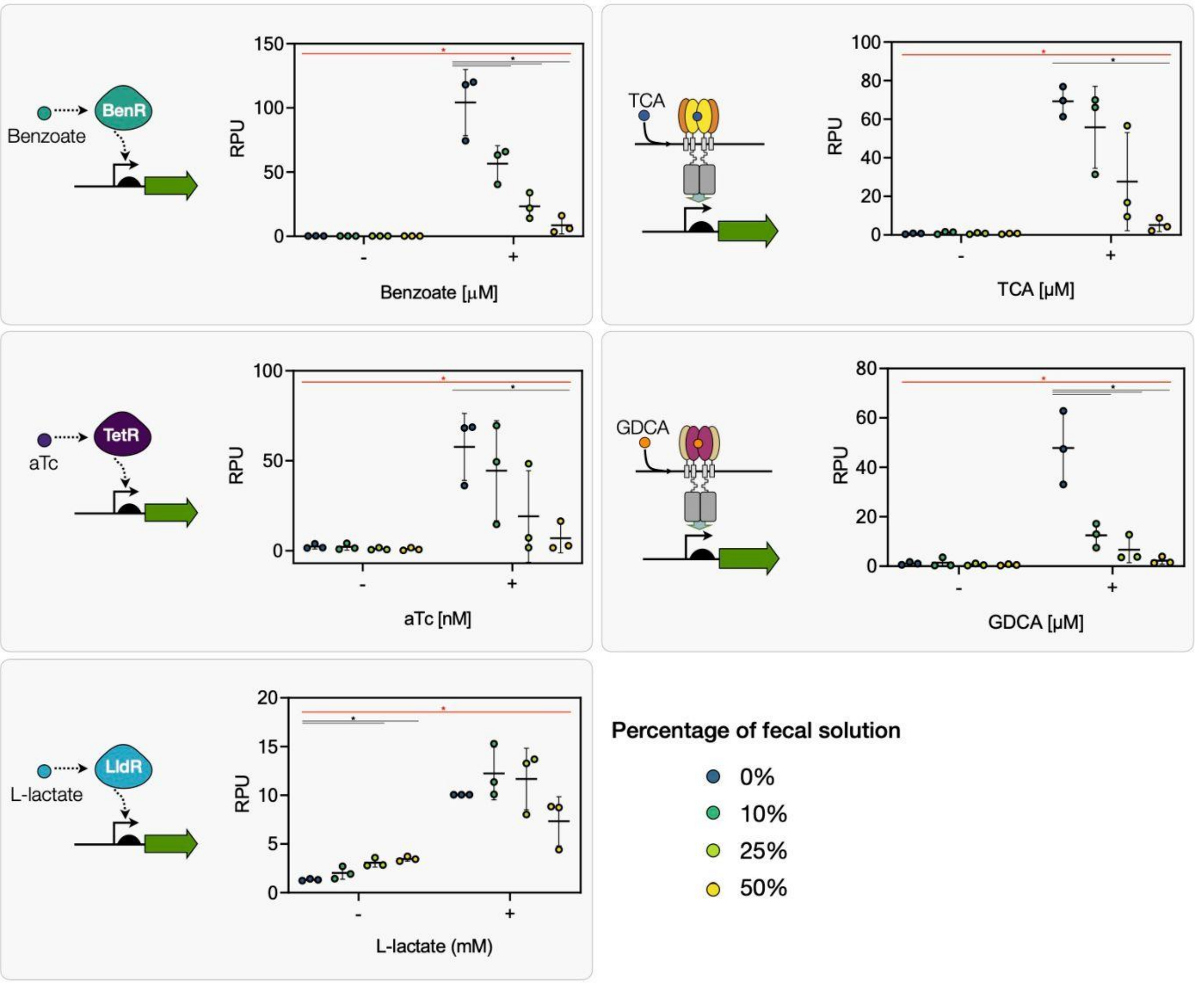

Supplementary figure S3. Matrix effect of pre-treated human feces on the performance of five different biosensors. Pre-treated samples of feces from three different patients (S1-S3, for BenR-pBEN, pALPAGA and TetR-pTET and S4-S6 for TcpP/TcpH and VrtA/VtrC) were used to evaluate the effect matrix on each biosensor performance. Three different dilutions of samples were used at final percentages; 10%, 25%, 50%. The relative promoter units (RPU) for each biosensor is shown. The geometric mean of two technical replicates for each patient sample performed on different days is plotted. The averages and standard deviations for these data are provided in supplementary excel data. Inducers; 100 µM benzoate, 200 nM aTc, 10 mM L-lactate, 100 µM TCA and 100 µM GDCA. * : P-value < 0.05 two-way ANOVA with Fisher's LSD multiple comparisons test. The asterisk represents significant differences between non-induced versus induced condition (red) and non-fecal solution versus different percentages of fecal solution (balck).

Supplementary figure S4

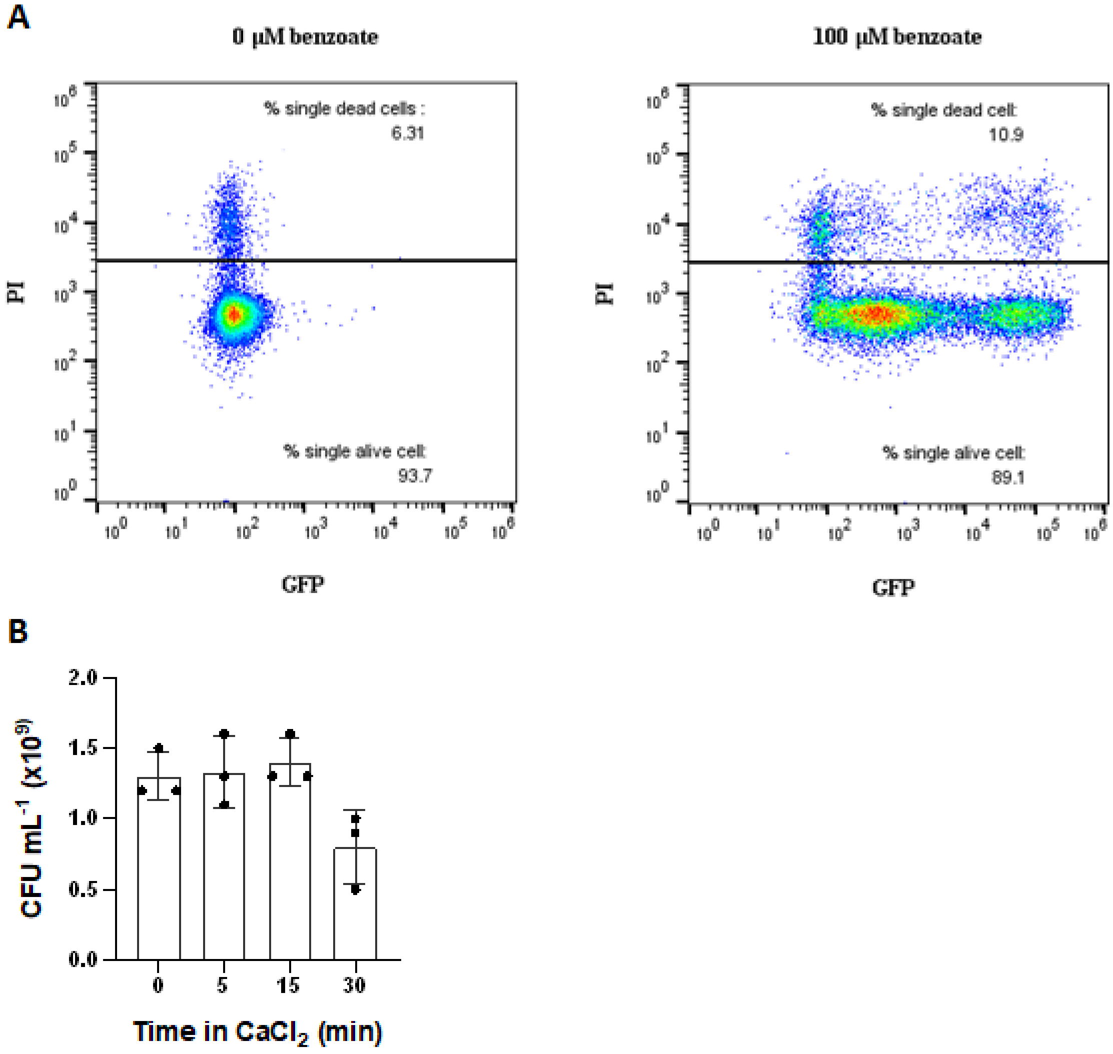

Supplementary Figure S4. Viability of BenR-pBEN biosensor encapsulated in alginate hydrogel beads. (A) Density plots of BenR-pBEN biosensor single cells retrieved from hydrogel beads, induced or not with 100 µM benzoate in 25µg/ml LB chloramphenicol after 16 h of incubation. Cells were subsequently stained with 0.01% propidium iodide (PI) for 15 min in the dark to assess cell death. Bacteria were recovered from hydrogel beads by dissolving them in a solution of 55 mM sodium citrate in PBS (pH=7.3) at 37 °C for 50 min with shaking at 190 rpm. Events above the horizontal black line are considered dead cells; this gate was determined by using heat-killed cells. (B) Colony forming units (CFU) of bacteria recovered from hydrogel beads after different incubation times in 5 w/v% $CaCl_2$ (alginate crosslinking step); 0, 5, 15 and 30 min. Cells were retrieved as mentioned above, then serial dilutions were placed in LB-agar 25µg/ml chloramphenicol plates and incubated at 37°C overnight. Bars represent the mean value of beads analyzed in triplicate. Error bars: ± SD

Supplementary figure S5

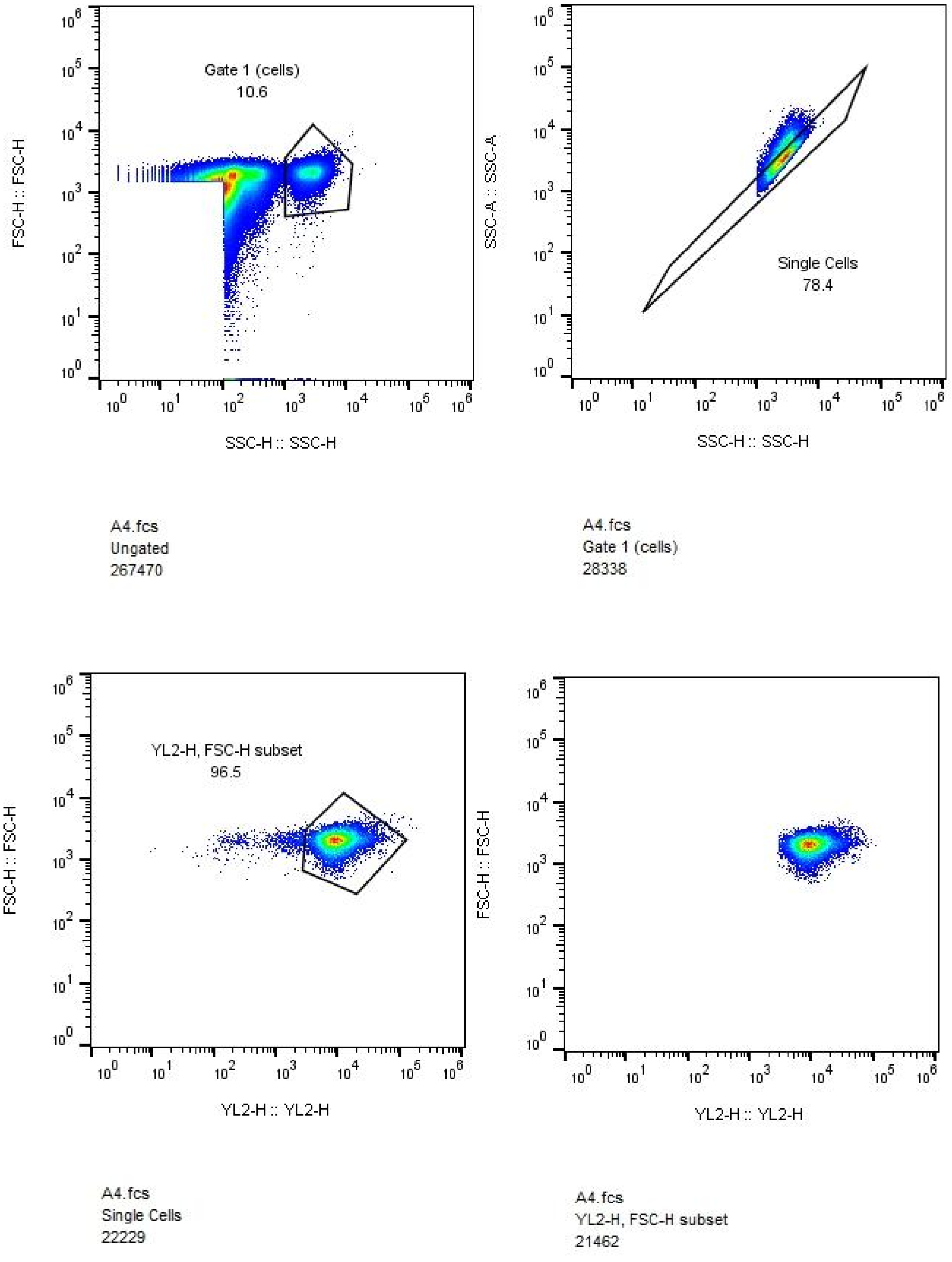

Supplementary Figure S5. Figure exemplifying the gating strategy. Gates were designed based on FSC-H vs SSC-H graphs to remove debris from the analysis and SSC-A vs SSC-H to doublet discrimination (lef and right upper panel). Cell subsets expressing the same fluorescent protein were selected with gates based on green, red and blue intensities (bottom panels).

Table S1. Bacterial biosensor used in this study

Bacterial Biosensor	Strain	Plasmid	ORI	Reference
**TcpP/H**	*E. coli* NEB10β	J64100 Chl^r	p15A	Chang et al., 2021
**VtrA/C**	*E. coli* NEB10β	J64100 Chl^r	p15A	Chang et al., 2021
**TetR-pTET**	*E. coli* DH5αZ1	pZ derivative Km^r	p15A	Lutz and Bujard, 1997
**LldR-pALPAGA**	*E. coli* DH5αZ1	pSB4K5 Km^r	pSC101	Zúñiga et al., 2021
**BenR-pBEN**	*E. coli* DH5αZ1	J64100 Chl^r	ColE1	Zúñiga et al., 2020

Chl^r, Km^r: Resistance to chloramphenicol and kanamycin, respectively.

Table S2. Bile acid and L-lactate levels in feces samples

Sample number	Total bile acids (μM)	L-lactate (mM)
**S1**	124	1.6
**S2**	68.6	0.1
**S3**	63.8	2.0
**S4**	29.6	1.1
**S5**	15.2	1.0
**S6**	13.5	0.6
**S7**	29	
**S8**	67	
**S9**	96.5	
**S10**	94	
**S11**	113.3	
**S12**	150.7	
**S13**	163.4	
**S14**	170	
**S15**	176	
**S16**	219	
**S17**	501	
**S18**	548	

Values were determined by enzymatic assays.

Table S3. Relative Percentage of Activity

% Of feces sample	10 %	25 %	50 %
**TcpPH**	82 ± 23	40 ± 36	7 ± 5
**VtrAC**	21 ± 6	11 ± 8	4 ± 2
**TetR-pTET**	50 ± 22	33 ± 34	12 ± 11
**LldR-pALPAGA**	120 ± 26	114 ± 36	72 ± 27
**BenR-pBEN**	54 ± 2	22 ± 9	8 ± 10

Average of Relative Percentage of Activity ($\overline{RPA}$) = 100-(∑(RPU_{iC}-RPU_{iS})*100/∑(RPU_{ic})) where RPU_{is} correspond to the fluorescence intensity in RPU in presence of both fecal solution (at different %) and the inductor, and RPU_{ic} correspond to the fluorescence intensity in RPU in control condition in presence of the inductor only.

Relative Percentage of Activity (RPA) = 100-[(RPU_{iC}-RPU_{iS})*100/(RPU_{ic})], where RPU_{is} corresponds to the fluorescence intensity in RPU in presence of both fecal solution (at different %) and the inductor, and RPU_{ic} corresponds to the fluorescence intensity in RPU in the control condition in presence of the inductor only. Mean values **±** SD are provided.

Protocol for feces processing and functional assessment of bacterial biosensors on feces.

Materials

- Copan Liquid Amies Elution Swab (ESwab®) Collection and Transport System (ESWABR1, Copan ITALIA S.p.A).
- Vortex
- Centrifuge
- Eppendorf tubes
- 0.45 μm pore size hydrophilic PVDF membrane (Millex-HV Syringe Filter, Millipore).
- Sterile syringe
- 96-well plate
- 2X concentrate LB

Procedure

Feces processing

1. Collect the samples from the patients using the ESwab collection system.
2. Homogenize by vortexing for 2 minutes.
3. Take the homogenized fecal solution and transfer in an eppendorf tube.
4. Centrifuge the samples at 4000 rpm for 10 min.
5. Recover the supernatant in a new eppendorf tube.
6. Filter the fecal solution by using a by using a 13 mm diameter sterile syringe filter with a 0.45 μm pore size hydrophilic PVDF membrane.

Functional characterization of bacterial biosensors

Day 1. Streak the bacterial biosensor from glycerol stock on LB agar plates and incubate at 37°C overnight.

Day 2. Pick three fresh colonies and inoculate into LB and grow at 37°C for 16 h.

Day 3. Dilute the fecal solution and the liquid culture in a 96-well plates as follow:

a. For 2 times-fold dilution: 75 μL 2X LB medium, plus 1.5 μL of biosensor culture, plus 3 μL of inducer adjusted at the needed concentration and 70.5 μL of the feces solution diluted in ESwab™ buffer to have a final volume of 150 μL.
b. For 4 times-fold dilution: 75 μL 2X LB medium, plus 1.5 μL of biosensor culture, plus 3 μL of inducer adjusted at the needed concentration and 15 μL of feces samples

plus 55.5 μL of ESwab™ buffer.
Incubate in 96-well plates, incubated at 37°C without shaking for 16 h.

Day 4. Mix the cells and dilute 100-times in 96-well plates and analyze them by flow cytometry.

Protocol for bacterial biosensor encapsulation and functional assessment on feces.

1. Encapsulation of biosensor in alginate hydrogel beads.

Materials

- 5 % w/v of alginate solution (Sigma-Aldrich A2033) in MilliQ water, autoclave sterilized.
- 5 % w/v CaCl2 solution (Sigma-Aldrich C1016)
- Black 384-well plate

Procedure

Day 1. Grow the cells in LB medium at 37 °C with shaking at 200 rpm for 16 h.

Day 2. Centrifuge the cells at 4000 rpm for 5 min and resuspended in fresh LB medium and adjust the OD at 4. Mix the cells with the alginate solution at 1:1 ratio to reach 2.5 % alginate. Drop 10 µL of this solution into a sterile $CaCl_2$ solution to form beads of 2 mm diameter for 5 min to perform the cross-linking.

2. Functional assessment of biosensor alginate beads on feces.

1. Incubate the bacterial alginate beads in a black 384-well plate with LB medium and fecal solutions as follow:
   For 2 times-fold dilution: 15 µL 2X LB medium, plus 15 µL of fecal solution and 1µL of inducer adjusted at the needed concentration.
2. Incubate at 37°C for 16 h without shaking.
3. Measure the fluorescence intensity and the bacterial optical density by using a plate reader.
